# Does the size of the neuroendocrine-carcinoma component determine the prognosis of gallbladder cancer?

**DOI:** 10.3389/fendo.2024.1217250

**Published:** 2024-07-22

**Authors:** Ya-Fei Hu, Jun-Ke Wang, Wen-Jie Ma, Hai-Jie Hu, Han-Fei Gu, Fei Liu, Tian-Run Lv, Si-Qi Yang, Yu-Shi Dai, Rui-Qi Zou, Yan-Wen Jin, Fu-Yu Li

**Affiliations:** ^1^ Department of Biliary Surgery, West China Hospital, Sichuan University, Chengdu, China; ^2^ Department of Biliary Disease Research Center, West China Hospital of Sichuan University, Chengdu, China

**Keywords:** gallbladder neuroendocrine carcinomas, gallbladder mixed adenoneuroendocrine carcinoma, gallbladder carcinoma, survival, neuroendocrine carcinoma

## Abstract

**Background:**

Gallbladder mixed neuroendocrine-non-neuroendocrine neoplasm generally consists of a gallbladder neuroendocrine tumor and a non-neuroendocrine component. The World Health Organization (WHO) in 2019 established a guideline requiring each component, both neuroendocrine and non-neuroendocrine, to account for a minimum of 30% of the tumor mass.

**Methods:**

Patients after surgery resection and diagnosed at microscopy evaluation with pure gallbladder neuroendocrine carcinoma (GBNEC), gallbladder mixed adeno-neuroendocrine carcinoma (GBMANEC, GBNEC≥30%), and gallbladder carcinoma mixed with a small fraction of GBNEC (GBNEC <30%) between 2010 and 2022 at West China Hospital of Sichuan University were collated for the analyses. Demographic features, surgical variables, and tumor characteristics were evaluated for association with patients’ overall and recurrence-free survival (OS and RFS).

**Results:**

The study included 26 GBNEC, 11 GBMANEC, 4 gallbladder squamous-cell carcinoma (GBSCC), and 7 gallbladder adenocarcinoma (GBADC) mixed with a small fraction of GBNEC. All patients had stage III or higher tumors (AJCC^8th^ edition). The majority of included patients (79.17%) underwent curative surgical resection (R0), with only ten patients having tumoral resection margins. In the analysis comparing patients with GBNEC percentage (GBNEC≥30% vs. GBNEC<30%), the basic demographics and tumor characteristics of most patients were comparable. The prognosis of these patients was also comparable, with a median OS of 23.65 months versus 20.40 months (P=0.13) and a median RFS of 17.1 months versus 12.3 months (P=0.24). However, patients with GBADC or GBSCC mixed with GBNEC <30% had a statistically significant decreased OS and RFS (both P<0.0001)) compared with GBNEC and GBMANEC. Patients with GBNEC who exhibited advanced tumor stages and lymphovascular invasion had a higher risk of experiencing worse overall survival (OS) and recurrence-free survival (RFS). However, a 30% GBNEC component was not identified as an independent risk factor.

**Conclusion:**

Patients with GBNEC were frequently diagnosed at advanced stages and their prognosis is poor. The 30% percentage of the GBNEC component is not related to the patient’s survival.

## 1 Introduction

Gallbladder neuroendocrine cancer (GBNEC) is a rare and aggressive disease that accounts for less than 0.2% of all gastrointestinal neuroendocrine cancers (1–3). Published medical literature reported that GBNEC had a more aggressive biological behavior and a poorer prognosis than other well-differentiated gallbladder neuroendocrine neoplasms (GBNEN) (4–8). The microscopy diagnosis of poorly differentiated neuroendocrine tumor cells serves as the cornerstone in the diagnosis of GBNEC (9, 10). Currently, for patients with resected GBNEC, surgical resection is the first-line treatment. The role of adjuvant therapy in the management of GBNEC is not well established.

Patients with gallbladder adenocarcinoma (GBADC) may have a tumor containing a GBNEC component on microscopy (11, 12). According to the 2019 World Health Organization (WHO) classification, if GBNEC components represent≥30% of the tumor mass, the mixed tumor is classified as gallbladder mixed adeno-neuroendocrine carcinoma (GBMANEC) (13). GBMANEC is a distinct subtype of gallbladder mixed neuroendocrine-non-neuroendocrine neoplasm (GBMiNEN), which is characterized by ≥30% GBNEC component of the tumor mass (9). Our institution has encountered cases where patients were diagnosed with GBADC or gallbladder squamous cell carcinoma with a small proportion of GBNEC (GBNEC<30%) within the tumor mass. These patients did not fulfill the diagnostic criteria for GBMiNEN as defined by the 2019 WHO classification (13). There is limited clinical evidence for these patients, as the disease incidence rate is very low.

The biological characteristics of the tumor determine the prognosis of the patient. Machairas N (14) and de Mestier L (15) concluded that in patients with mixed gastrointestinal neoplasms, the prognosis was related to the most aggressive neoplastic elements, regardless of their extension in the tumor. The WHO 2019 has defined GBMiNEN using a cut-off value of 30% (13). However, this threshold is considered inappropriate and lacks clinical support (9, 16). The aggressiveness of the tumor may not be directly related to the percentage of aggressive GBNEC elements. Published medical literature found that tumors with even a small proportion of primary GBNEC lesions may have a higher risk of developing deep infiltration or distant metastases than those without (9, 14, 16, 17). Unfortunately, due to low -incidence rate, clinical management for these patients were largely unknown. Therefore, we aim to investigate the clinicopathological features and prognosis of pure GBNEC (i.e.only the GBNEC component of the tumor mass), GBMANEC (i.e. GBNEC ≥30%) and GBADC or GBSCC, both mixed with a small fraction of GBNEC (GBNEC <30% of the tumor mass). The study also determined the factors associated with the long-term prognosis in these patients.

## 2 Material and methods

### 2.1 Study population

The research was led Department of Hepatobiliary Surgery, West China Hospital of Sichuan University. Patients after surgical resection and being pathologically diagnosed with GBNEC, GBMANEC, and GBC mixed with a small fraction of GBNEC (<30%) between 2011 and 2023 were collated for the retrospective analyses. The GBNEC and GBMANEC diagnostic criteria were based on the WHO 2019 classifications (18, 19). All included patients have been pathologically confirmed by two independent and experienced pathologists.

The study only included patients who met all the following criteria: (i) primary tumor originating from the gallbladder, (ii) diagnosis of GBNEC, and GBMANEC component were based on pathological and immunohistochemical results according to the WHO 2019 classification (19), and (iii) cases with comprehensive medical records. Cases that met any of the following criteria were excluded: (a) Neuroendocrine carcinomas originating from extrahepatic or cystic ducts or metastases from organs such as the liver, lung, or pancreas; (b) cases with incomplete medical records or missing data; (c) cases with well-differentiated neuroendocrine neoplasms (defined as a Ki-67 of <20%, according to the WHO 2019 classification); and (d) patients with distant metastases (M1).

**Figure 1:**
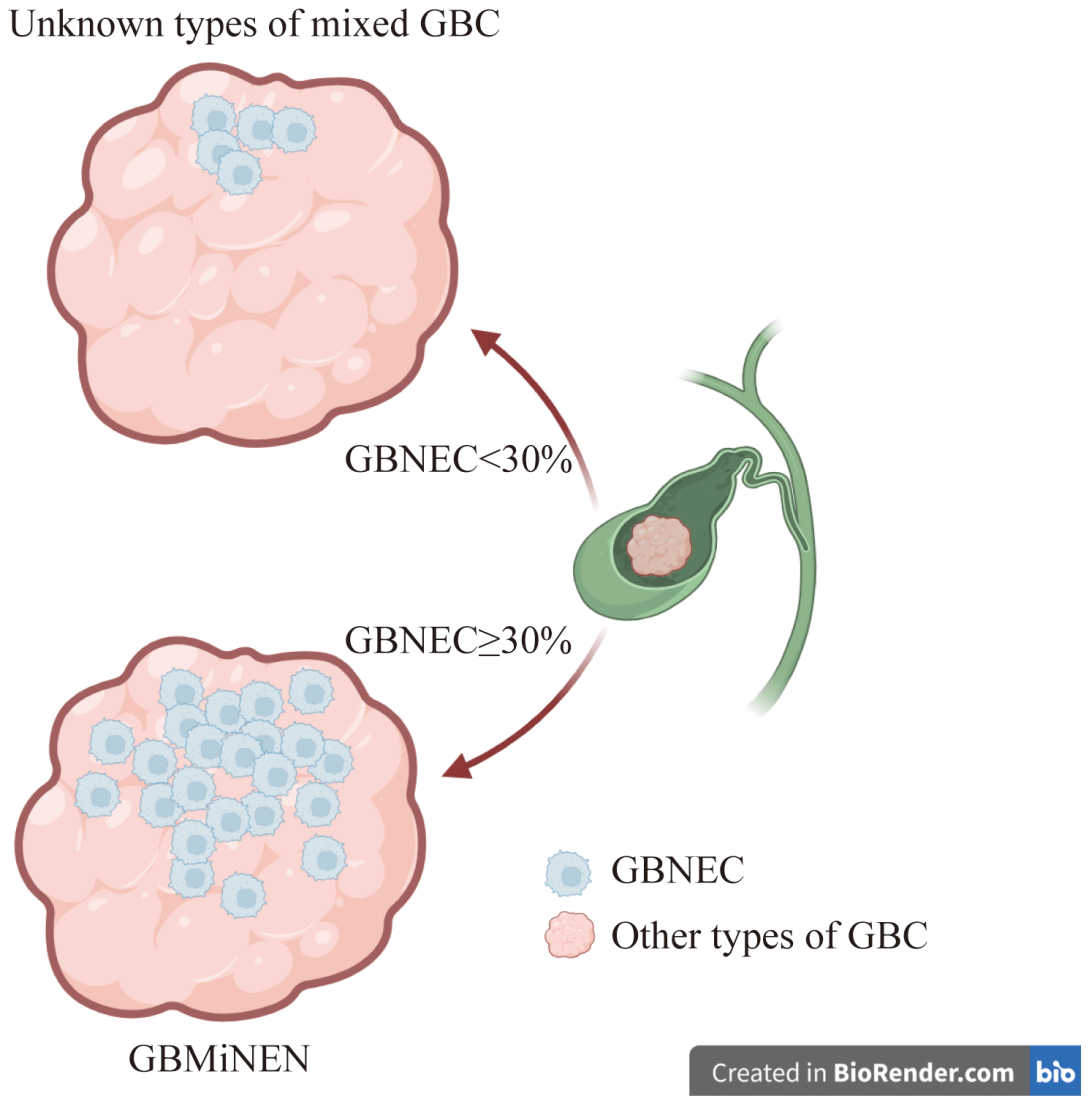
Description and definition of gallbladder cancer mixed with GBNEC component.

**Figure 2:**
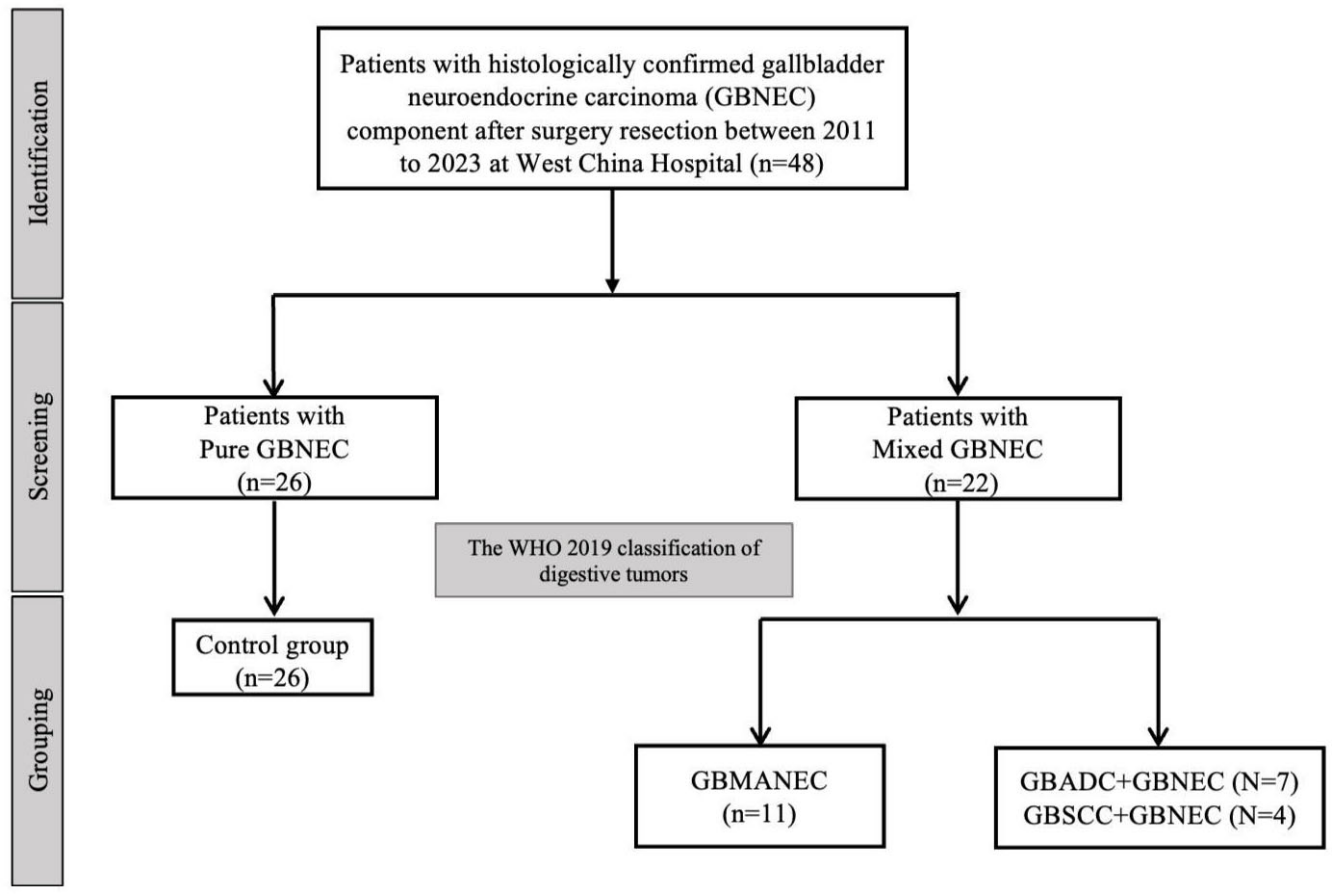
The study flow diagram.

A total of 26 cases of pure GBNEC (tumor mass consisted of GBNEC component only), eleven cases of GBMANEC, 4 cases of GBSCC mixed with a small fraction of GBNEC (GBNEC<30%), and 7 cases of GBADC mixed with a small fraction of GBNEC were included (GBNEC<30%). These enrolled patients were divided into GBNEC≥30% and GBNEC <30% cohorts according to the GBNEC percentage of the tumor mass (**Figure 1**). Patients with GBADC/GBSCC mixed with a small fraction of GBNEC were selected in the GBNEC <30% cohorts as the experimental group (N=11), while patients with pure GBNEC and GBMANEC were used as the control group (N=38). The study flow diagram is provided in **Figure 2**.

Neuroendocrine markers, including synaptophysin (Syn), chromogranin (CgA), CD56, and Ki-67 staining index, were performed in most included cases. The following clinical information: age at diagnosis, gender, laboratory examination outcomes before the operation, type of resection (RC and ERC), and tumor stages (based on the eighth American Joint Committee on Cancer (AJCC 8^th^) staging system for biliary tract cancers) (20), etc. were also collected and are presented in **Table 1**.

**Table 1:** Clinicopathologic characteristics of groups with GBNEC<30% and GBNEC≥30%.

Variable	Overall (N=48)	GBNEC<30% (N=11)	GBNEC≥30% (N=37)	*p-value*
Age, years, Mean ± SD	55.35 ± 10.08^a^	57.18 ± 12.66^a^	54.81 ± 9.32^a^	0.499
Age, years				
<60y	31 (64.58%)	6 (54.5%)	25 (67.6%)	0.664
≥60y	17 (35.42%)	5 (45.5%)	12 (32.4%)	
SEX, N (%)				
Female	32 (66.67%)	5 (45.5%)	27 (73%)	0.182
Male	16 (33.33%)	6 (54.5%)	10 (27%)	
ALT, IU/L	90.99 ± 124.17^a^	41.00 (24.00 to 135.00) ^b^	27.00 (14.00 to 75.00) ^b^	0.411
AST, IU/L	65.08 ± 89.23^a^	28.00 (22.00 to 92.50) ^b^	23.00 (18.00 to 51.00) ^b^	0.326
TB, μmol/L	45.65 ± 94.72^a^	15.50 (11.85 to 32.70) ^b^	11.40 (8.50 to 14.40) ^b^	0.050
WBC, 10^9^/L	7.44 ± 3.57^a^	7.45 (6.49 to 11.61) ^b^	6.53 (5.17 to 8.29) ^b^	0.121
AFP, ng/ml	5.52 ± 11.66^a^	3.21 (2.35 to 4.37) ^b^	3.54 (2.40 to 4.93) ^b^	0.652
CA125, U/mL	36.77 ± 46.81^a^	20.89 (16.63 to 49.34) ^b^	24.19 (16.63 to 39.20) ^b^	0.703
CA199, U/mL	89.56 ± 193.01^a^	10.04 (8.36 to 25.15) ^b^	18.41 (8.47 to 41.58) ^b^	0.300
CA 19-9≥37.0 U/mL	20 (41.67%)	5 (45.5%)	15 (40.5%)	1.000
CA 125≥35.0 U/mL	11(22.92%)	3 (27.3%)	8 (21.6%)	1.000
Adjuvant radio-chemotherapy, N (%)	8 (16.67%)	3 (27.3%)	5 (13.5%)	0.539
Ki-67 index	53.60 ± 22.44^a^	59.45 ± 26.12^a^	51.86 ± 21.32^a^	0.330
CgA (+), N (%)	42 (87.50%)	7 (63.6%)	35 (94.6%)	**0.027**
Syn (+), N (%)	37 (77.08%)	9 (81.8%)	28 (75.7%)	0.986
CD56 (+), N (%)	31 (64.58%)	9 (81.8%)	22 (59.5%)	0.316
pTNM stages^c^, N (%)				
IIIA	16 (33.33%)	5 (45.5%)	11 (29.7%)	0.357
IIIB	9 (18.75%)	3 (27.3%)	6 (16.2%)	
IVA	6 (12.50%)	0 (0%)	6 (16.2%)	
IVB	17 (35.42%)	3 (27.3%)	14 (37.8%)	
Type of resection, N (%)				
Extended Radical cholecystectomy, N (%)	36 (75.00%)	**5 (45.5%)**	**31 (83.8%)**	**0.029**
Radical cholecystectomy, N (%)	12 (25.00%)	6 (54.5%)	6 (16.2%)	
Operative duration, min	268.65 ± 71.98^a^	265.91 ± 82.67 ^a^	269.46 ± 69.73 ^a^	0.888
Estimated blood loss, ml	350.00 ± 220.73^a^	250.00 (250.00 to 325.00) ^b^	300.00 (250.00 to 450.00) ^b^	0.425
Resection margins, N (%)				
R0	38 (79.17%)	10 (90.9%)	28 (75.7%)	0.503
R1	10 (20.83%)	1 (9.1%)	9 (24.3%)	
Postoperative morbidity ^d^, N (%)				
I	5 (10.42%)	0 (0%)	5 (13.5%)	0.421
II	34 (70.83%)	9 (81.8%)	25 (67.6%)	
IIIA	9 (18.75%)	2 (18.2%)	7 (18.9%)	
Duration of hospital stay, days	15.42 ± 2.66^a^	14.89 ± 2.34^a^	15.39 ± 2.83^a^	0.593
90-day mortality, N (%)	1 (9.1%)	1 (9.1%)	0 (0%)	0.515
Distant metastasis, N (%)				
Liver	42 (87.50%)	10 (90.9%)	32 (86.5%)	0.778
Peritoneum	3 (6.25%)	1 (9.1%)	2 (5.4%)	
Bone	1 (2.08%)	0 (0%)	1 (2.7%)	
Lt. subclavian lymph node	2 (4.17%)	0 (0%)	2 (5.4%)	
Microscopic perineural invasion, N (%)	15 (31.25%)	3 (27.3%)	12 (32.4%)	1.000
Lymph vascular invasion, N (%)	27 (56.25%)	8 (72.7%)	19 (51.4%)	0.364
Positive lymph nodes, N (%)	28 (58.33%)	6 (54.5%)	22 (59.5%)	1.000

a data presented as Median ± SD.

b data presented as Median (IQR).

c All staging was based on the 8th edition of the AJCC TNM staging system.

d Complications were graded by Clavien-Dindo≥III classification grades.

GBSCC, gallbladder squamous cell carcinoma; GBADC, gallbladder adenocarcinoma; GBNEC, gallbladder neuroendocrine cancer; GBMANEC, gallbladder mixed adeno-neuroendocrine carcinoma; RC, Radical cholecystectomy; ERC, Extended radical cholecystectomy; CA125, carbohydrate antigen 125; CA19-9, carbohydrate antigen 19-9; TB, Total Bilirubin, AST, Aspartate Aminotransferase; WBC, Write Blood Cell; AFP, Alpha-Fetoprotein, ALT, Alanine Aminotransferase; CgA, chromogranin A; NEC, neuroendocrine carcinoma; Syn, synaptophysin; pTNM, pathogenic tumor node metastasis.

### 2.2 Definitions and follow-up

The Clavien-Dindo classification system was used to categorize all postoperative complications grades. Severe postoperative morbidity was defined as patients with postoperative mortality greater than III. The postoperative 90-day mortality was defined as operative-related death within 90 days after surgery. Cancer antigen 19-9 (CA 19-9; also known as carbohydrate antigen 19-9) is used to help differentiate between cancer of the pancreas and other conditions, as well as to monitor treatment response and recurrence. The normal range of CA 19-9 is between 0 and 37 U/mL (units/milliliter). In this study, patients were grouped into CA 19-9≥37.0 U/mL vs. CA 19-9 <37.0 U/mL (Elevated vs. Normal). The normal range of Carbohydrate antigen 125 (CA125) is between 0 and 35 U/mL (units/milliliter).In this study, patients were grouped into CA 125≥35.0 U/mL vs. <35.0 U/mL (Elevated vs. Normal).

After surgery, patients were re-evaluated monthly for the first year and then every 3 months for the following 2 years. To evaluate the surgical results and to detect possible recurrence, physical examinations, laboratory tests, and abdominal CT or MRI scans were performed. In cases where the patient could not attend the scheduled follow-up appointments, telephone interviews were conducted. All patients’ follow-up duration was defined as the date of diagnosis to the last examination date or lost follow-up. Overall survival (OS) was considered as the interval from the date of surgery to the date of death or the most recent follow-up. Recurrence-free survival (RFS) was considered as the interval from the date of surgery to recurrence or metastasis or last follow-up if recurrence did not occur during follow-up.

### 2.3 Surgical methods

For patients with T3N0M0 stages of the tumor (tumor liver invasion <2cm), radical cholecystectomy (RC) combined with liver resection such as IVb+V segment resection and lymph node dissection (LND) may be considered; for those patients with T3N1M0, considering that cancer cells may have metastasized along the lymphatic or Glisson system to the entire right half of the liver, in our medical center, we required right hemi hepatectomy or right anatomical hepatectomy combined with a comprehensive LND. For selected stage IV GBC (T4N0MO, T4N1M0, or TxN2M0) with a tumor invading the duodenum, stomach, pancreas, or colon if an R0 resection can be safely achieved, open surgery with multiple organ resection is adopted for these patients. The para-aortic lymph nodes (No. 16) should be rapidly frozen if there was preoperative imaging or intraoperative suspicion of tumor invasion of the surrounding tissues, such as the liver, or other adjacent organs. The presence of positive tumoral lymph nodes at level No.16 may indicate that radical surgery should be avoided. All patients included in this study underwent D2 LND, which included the celiac artery, common hepatic artery, retro-pancreatic, and hepatoduodenal ligament lymph nodes. Extended radical cholecystectomy (ERC) included patients who underwent right hemi hepatectomy, right anatomical hepatectomy, multiple organ resection, or extended LND.

### 2.4 Ethical approval

This study was conducted in accordance with the Declaration of Helsinki and in compliance with the study protocol and ethical guidelines for medical and health research involving human subjects. The study was approved by the institutional review committee of West China Hospital, Sichuan University (approval code 2021-445). A summary of the protocol of the study is available on the website of the hospital.

### 2.5 Statistical analysis

Tumor parameters and patient demographics were presented as mean (SD) for parametric continuous data and median (IQR) for non-parametric data. Categorical data were presented as percentages (N, %). Fisher’s exact test, chi-squared test, or independent t-test were used to detect significant differences between groups, as appropriate.

OS and RFS were estimated using the Kaplan-Meier method. Univariate analysis was performed to evaluate the predictive value of clinicopathological factors. In the univariate analysis, characteristics showing significance at P < 0.05 underwent multivariate analysis to uncover independent variables associated with OS and RFS. Hazard ratios (HR) with 95% confidence intervals (CI) were calculated from the multivariable analysis. Patients were stratified according to tumor diagnosis or the percentage of GBNEC (GBNEC ≥30% vs. <30%) for subgroup analyses.

All statistical tests performed were two-tailed, and a P value of less than 0.05 was considered statistically significant. All statistical analyses and graphs were performed using R software version 4.1.0.

## 3 Results

### 3.1 Patients and tumor characteristics

**Table 1** summarizes the characteristics of the patients included in the study. There were 48 patients (median [SD] age, 55.35 [10.08] years), consisting of 26 patients (54.2%) with pure GBNEC, 11 patients (22.9%) with GBMANEC, and 4 cases of GBSCC and 7 cases of GBADC mixed with a small fraction of GBNEC (8.3% and 14.6%, respectively). The gender distribution of the patients was 16 males (33.3%) and 32 females (66.7%). All patients were diagnosed with stage III or higher tumors (AJCC 8th edition) according to postoperative pathology. Most patients (38 of 48, 79.17%) underwent curative surgical resection (R0), while ten patients had positive tumoral resection margin (R1).

In subgroup analyses of GBNEC percentage (GBNEC≥30% and GBNEC<30%), most patients’ basic demographics and tumor characteristics were comparable, except for patients in the GBNEC<30% group, who had a lower rate of ERC (45.5% vs. 83.8%, P=0.029). Immunohistochemical staining analysis revealed that most tumors were positive for CgA (87.5%), followed by Syn (77.1%) and CD56 (64.6%). In addition, the median Ki-67 index was calculated for the whole cohort (median [SD], 53.60[22.44]). Patients with GBNEC≥30% and GBNEC<30% also had similar immunohistochemical staining results, except for a lower Syn positive rate in the GBNEC<30% group (63.6% vs. 94.6%, P=0.027).

### 3.2 Long-term outcomes

**Figure 3:**
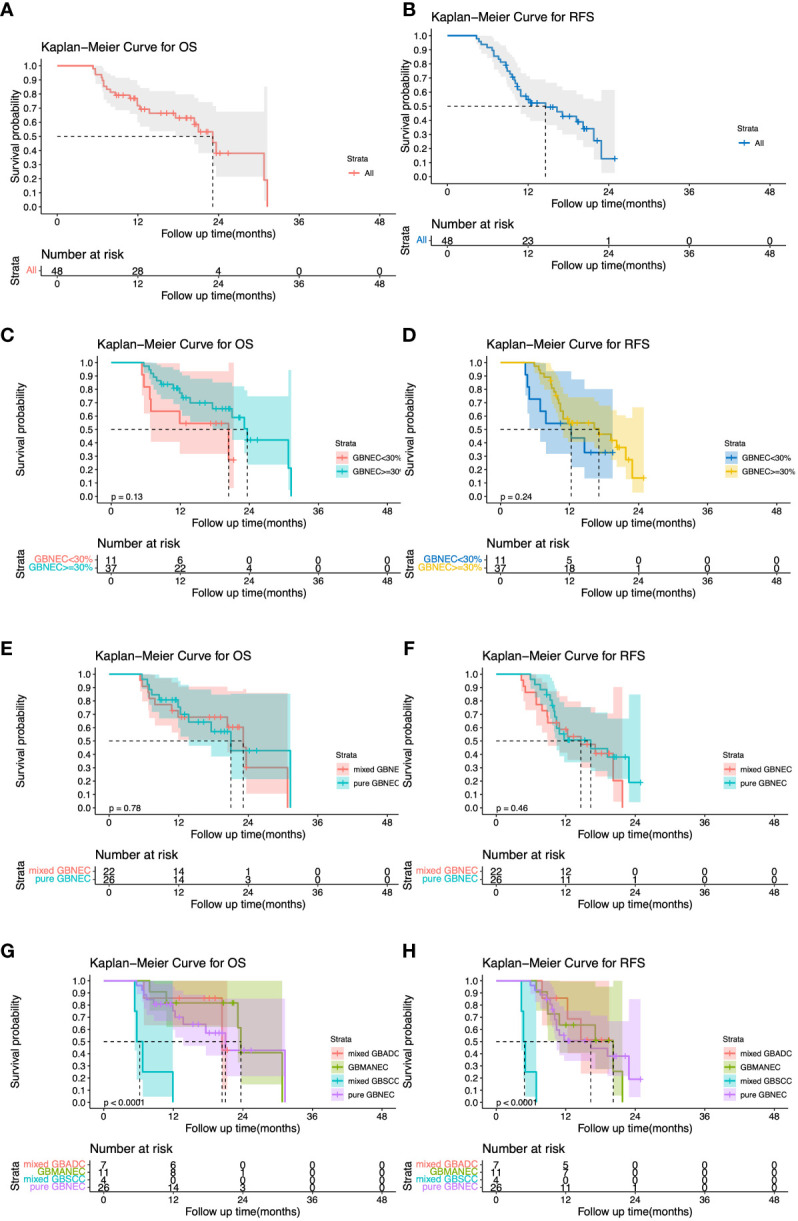
Overall and Recurrence-free survival (OS and RFS) for 48 patients with gallbladder neuroendocrine carcinoma (GBNEC) component. **(A)** OS for 48 patients with GBNEC component; **(B)** RFS for 48 patients with GBNEC component; **(C)** OS for patients with GBNEC component<30% (N=11) vs. ≥30% (N=37) **(D)** RFS for patients with GBNEC component<30% (N=11) vs. ≥30% (N=37); **(E)** OS for patients with pure GBNEC (N=26) vs. mixed GBNEC (N=22); **(F)** RFS for patients with pure GBNEC (N=26) vs. mixed GBNEC (N=22); **(G)** OS for patients with different types of gallbladder cancer; **(H)**. RFS for patients with different types of gallbladder cancer.

All patients in the GBNEC component <30% group experienced a recurrence after surgery. The most frequent site was the liver (90.9%) followed by the peritoneum. Most patients in the GBNEC component ≥30% group also had a recurrence in the liver (86.5%), followed by the peritoneum (5.4%), Lt. subclavian lymph node (5.4%), and bone (2.7%). The median overall survival (mOS) and recurrence-free survival (mRFS) for all 48 patients with GBNEC components were 23.13 months and 14.60 months, respectively, as shown in **Figures 3A, B**. The prognosis of patients with GBNEC≥30% and GBNEC<30% was comparable, with a median OS of 23.65 months versus 20.40 months (P=0.13) and a median RFS of 17.1 months versus 12.3 months (P=0.24), as is shown in **Figures 3C, D**. No significant differences were observed between patients with pure GBNEC and those with mixed GBNEC (GBMANEC, GBSCC/GBADC mixed with GBNEC<30%) in terms of mOS (21.00 versus 23.13 months, P=0.78) and mRFS (16.30 versus 14.60 months, P=0.46) (**Figures 3E, F**). However, a significant prognostic difference was found in OS of patients with pure GBNEC, GBMANEC, GBADC mixed with a small fraction of GBNEC, and GBSCC mixed with a small fraction of GBNEC, with mOS of 21.00, 23.65, 20.40, and 6.205 months, respectively (P<0.0001). The mRFS among patients with pure GBNEC, GBMANEC, and GBSCC mixed with a small fraction of GBNEC were also different. The mRFS periods for these groups were 16.30, 20.20, and 4.855 months, respectively (P<0.0001) (**Figures 3G, H**).

### 3.3 Risk factors of patients’ long-term overall survival and recurrence-free survival

Univariate Cox analysis revealed that advanced tumor pathological AJCC staging (P<0.0001), presence of microscopic perineural invasion (P=0.005), lymphovascular invasion (P=0.001), with positive lymph nodes (P=0.001) were significantly associated with decreased OS. However, the percentage of GBNEC in the tumor mass (≥30% versus <30%) was not found to be statistically associated with OS (P=0.141) or RFS (P=0.248). The tumor diagnosis mixed with GBNEC or pure GBNEC also had no significant impact on patients’ OS (P=0.782) and RFS (P=0.464). Multivariable analysis showed that advanced pathological AJCC staging (HR=2.76, 95%CI (1.13-6.74), P=0.025) and tumor presence of lymphovascular invasion (HR=8.72,95%CI (2.02-37.58), P=0.004) were independent risk factors for worse OS (**Table 2**). In addition, univariable analyses of RFS for included patients found type of resection (RC vs. ERC) (P=0.056), surgical resection margins (R1 vs. R0) (P=0.001)), advanced tumor pathological AJCC staging (P<0.0001), tumor presence of microscopic perineural invasion (P=0.001), lymphovascular invasion (P<0.0001), with positive lymph nodes (P=0.001) were associated with inferior RFS. While after multivariable analysis we found advanced pathological AJCC staging (HR=4.25,95%CI (1.95-9.26), P<0.0001) and tumor presence of lymphovascular invasion (HR=3.34, 95%CI (1.12-9.94), P=0.031) were independently associated with worse RFS (**Table 3**).

**Table 2:** Univariable and multivariable Cox regression analyses of predicting overall survival for included patients with resected GBNEC component.

Variables	HR Comparison	UV HR (95% CI)	UV *P*	MV HR (95% CI)	MV *P*
Age group	≥60y vs. <60y	1.69 (0.7-4.07)	0.239		
Sex	Male vs. Female	1.25 (0.46-3.37)	0.661		
CA125 group^a^	Elevated vs. Normal	1.11 (0.4-3.07)	0.839		
CA19-9 group^b^	Elevated vs. Normal	0.35 (0.13-0.92)	0.034	0.43 (0.14-1.29)	0.131
Adjuvant radio-chemotherapy	Yes vs. No	0.48 (0.11-2.05)	0.319		
Tumor diagnosis	pure GBNEC vs. mixed GBNEC	0.89 (0.37-2.1)	0.782		
GBNEC percentage classification	≥30% vs. <30%	0.48 (0.18-1.28)	0.141		
Type of resection	RC vs ERC	0.41 (0.12-1.43)	0.164		
Resection margins	R1 vs. R0	1.85 (0.57-6.03)	0.305		
TNM stages	IIIA vs. IIIB vs. IVA vs. IVB	3.82 (1.97-7.41)	<0.0001	2.76 (1.13-6.74)	0.025
Microscopic perineural invasion	Yes vs. No	4.41 (1.56-12.45)	0.005	0.34 (0.1-1.19)	0.090
Lymphovascular invasion	Yes vs. No	6.47 (2.09-20.1)	0.001	8.72 (2.02-37.58)	0.004
Positive lymph nodes	Yes vs. No	8.36 (2.34-29.84)	0.001	3.84 (0.7-20.89)	0.120

RC, Radical cholecystectomy; ERC, Extended radical cholecystectomy; SC, Simple cholecystectomy; GBNEC, gallbladder neuroendocrine carcinoma; mixed GBNEC, gallbladder cancer mixed with any percentage of GBNEC; CI, confidence interval; HR, hazard ratio; MV, multivariable; UV, univariable; P, P-value; CA125 group^a^: carbohydrate antigen 125 (CA 125≥35.0 U/mL vs. <35.0 U/mL); CA19-9 group^b^: carbohydrate antigen 19-9 (CA 19-9≥37.0 U/mL vs. <37.0 U/mL).

**Table 3:** Univariable and multivariable Cox regression analyses of predicting Recurrence-free survival for included patients with resected GBNEC component.

Variables	HR Comparison	UV HR (95% CI)	UV *P*	MV HR (95% CI)	MV *P*
Age group	≥60y vs. <60y	1.48 (0.69-3.17)	0.314		
Sex	Male vs. Female	1.60 (0.76-3.38)	0.213		
CA125 group^a^	Elevated vs. Normal	0.73 (0.29-1.83)	0.507		
CA19-9 group^b^	Elevated vs. Normal	0.48 (0.22-1.04)	0.064		
Adjuvant radio-chemotherapy	Yes vs. No	0.41 (0.12-1.37)	0.146		
Tumor diagnosis	pure GBNEC vs. mixed GBNEC	0.76 (0.36-1.6)	0.464		
GBNEC percentage classification	≥30% vs. <30%	0.6 (0.25-1.43)	0.248		
Type of resection	RC vs ERC	0.35 (0.12-1.03)	0.056	2.28 (0.5-10.35)	0.286
Resection margins	R1 vs. R0	3.95 (1.7-9.16)	0.001	0.57 (0.18-1.74	0.320
TNM stages	IIIA vs. IIIB vs. IVA vs. IVB	2.84 (1.88-4.27)	<0.0001	4.25 (1.95-9.26)	<0.0001
Microscopic perineural invasion	Yes vs. No	4.29 (1.83-10.05)	0.001	0.60 (0.20-1.84)	0.374
Lymphovascular invasion	Yes vs. No	5.13 (2.07-12.73)	<0.0001	3.34 (1.12-9.94)	0.031
Positive lymph nodes	Yes vs. No	4.78 (1.97-11.6)	0.001	0.56 (0.14-2.22)	0.411

RC, Radical cholecystectomy; ERC, Extended radical cholecystectomy; SC, Simple cholecystectomy; GBNEC, gallbladder neuroendocrine carcinoma; mixed GBNEC, gallbladder cancer mixed with any percentage of GBNEC; CI, confidence interval; HR, hazard ratio; MV, multivariable; UV, univariable; P, P-value; CA125 group^a^: carbohydrate antigen 125 (CA 125≥35.0 U/mL vs. <35.0 U/mL); CA19-9 group^b^: carbohydrate antigen 19-9 (CA 19-9≥37.0 U/mL vs. <37.0 U/mL).

## 4 Discussion

In this study, we included patients with pure GBNEC, GBMANEC, and GBC mixed with a small fraction of GBNEC (GBNEC component of the tumor mass<30%) to investigate the clinicopathological features and long-term prognosis difference of these neoplasms, and we noticed patients with GBC mixed with a small fraction of GBNEC (GBNEC<30%) showed a prognosis as dismal as patients with pure GBNEC and/or GBMANEC (GBNEC component of the tumor mass≥30%) in the present study.

Epithelial neoplasms with co-existing neuroendocrine and non-neuroendocrine histology (each representing from 1% to 99% of the tumor mass) have been described in almost all organs (21). These mixed neuroendocrine/non-neuroendocrine neoplasms have different definitions over time. In 2010, mixed neoplasms of the gastrointestinal and pancreatic tract containing neuroendocrine and non-neuroendocrine components in at least 30% of the tumor mass were classified separately by the World Health Organization and named MiNEN (19, 22, 23). In 2017, although the WHO also renamed MANEC as MiNEN which expanded the diagnostic range of mixed neuroendocrine tumors, the 30% threshold for each component has been maintained (18, 24). The rationale behind the 30% threshold is that a lesser-represented tumor component is unlikely to influence the biological behaviors of the whole neoplasm. However, the threshold was not supported by any clinical evidence of its relevance or significance to patients’ prognosis.

GBNEC have an aggressive biological behavior and inferior prognosis than other well-differentiated gallbladder neuroendocrine neoplasms or GBADC (25, 26). Lee et al. (27) included 34 cases of GBNECs and found that compared with stage-matched GBADCs both 1- and 3-year OS rates of patients with GBNEC were poorer than patients with GBADC (P < 0.001). Besides, clinical studies pointed out that the prognosis of GBMANEC was also worse than that of pure GBADC (4, 5, 17, 27–29). Thus, they suggested that the prognosis of digestive mixed neoplasm was determined by the most aggressive tumor component (9, 16). Simultaneously, gastric NECs and (or) gastric MANECs were found worse prognoses and more frequent recurrence than those with gastric ADC in a multicenter analysis (30). Unfortunately, multicenter large-sample studies comparing the long-term prognosis of pure GBNEC, GBMANEC, and GBADC are lacking due to their low incidence rate. Most analyses of GBMiNEN were case reports or series. Costa et al (31) published a literature review in 2021. They reported that there were only 24 cases of GBMiNEN. Although most research results support that the biological behavior of mixed neoplasms is determined by the most aggressive tumor component, whether the different proportions of these components will affect the prognosis of these GBMiNEN patients has not been reached consensus. In fact, some scholars of the gastric neopalsm have pointed out that tumor components’ aggressiveness may not be associated with these elements’ percentage (30). However, for gallbladder mixed neoplasm, this information is limited. After searching, we found 2 cases of GBADC mixed with a small fraction of NEC (16, 32). Both authors reported that GBADC mixed with a small fraction of GBNEC (GBNEC component ≤30%), which did not meet the criteria for GBMiNEN, showed rapid progress in a few months. Meanwhile, the accuracy of the WHO’s 30% threshold cannot be verified because we found no clinical studies presented the value of GBNEC components proportion of in the GBMiNEN tumor mass. In this study, we observed comparable survival duration for patients with pure GBNEC, GBMANEC, and those of GBADC mixed with a small fraction of GBNEC. In addition, we also found that the OS of patients with GB-SCC mixed with a small fraction of GBNEC was significantly poorer than that of GBNEC, GBMANEC, and GB-ADC mixed with a small fraction of GBNEC. Thus, our outcomes were not in favor of a 30% neuroendocrine component as a criterion to stratify GBNEC patients with different prognoses.

Which factors can be used to differentiate GBNEC patients’ prognosis other than 30% neuroendocrine component? Unfortunately, this question has not been answered in previous clinical investigations of GBNEC. However, a large cohort of clinical studies of gastrointestinal NEC has shown that NEC is a malignant component with a poor prognosis and there is no significant relationship with the proportion of NEC components (30, 33). Our results also have comparable findings. We noticed there is no significant OS differences between patients with GBNEC ≥30% and those with GBNEC <30%. Besides, patients with GB-SCC mixed with a small fraction of GBNEC (GBNEC<30% of the tumor mass) had the most inferior survival prognosis. The presence of highly malignant components of the tumor mass plays a key role in the patient’s long-term survival.

GBNEC is aggressive and may require extensive surgery resection to obtain a negative margin. Some studies supported aggressive treatment including extensive surgical resection and adjuvant radio-chemotherapy may be necessary for patients with GBNEC (11, 12, 29, 34). However, there were no clinical guidelines for patients with GBNEC or GBMiNEN, most treatment methods followed that of GBADC. In a recent study, Liu (3) and colleagues identified the mutation landscape associated with GBNEC, and their findings supported specific pathogenesis features of GBNEC. Thus, we hypothesize that the treatment of GBNEC should be different from GBADC. Previous studies have reported different findings. Lin (34) et al. found a GBMANEC case and suggested that a combination of surgery, ACT, and somatostatin treatment could lead to improved survival. However, Zhang et al. (29) concluded that extensive surgical resection remains the only key treatment for patients with GBNEC. In other studies, the benefits of curative resection for GBNEC were not clarified. We hypothesize this may be due to the aggressive nature of GBNEC that most patients were diagnosed in late stages, thus the patients may experience recurrence soon after surgical resection.

The study had several limitations. First, our patient data were collected from a referral medical center and thus may have introduced selection and information loss biases. Some late-stage patients were not included in the queue due to losing the opportunity for surgery. Additionally, the long follow-up span may result in poor compliance and follow-up biases, which could have affected the timely recording of specific survival outcomes for the included patients. Third, differences in patients’ living standards could have also introduced biases related to the prognosis. Fourth, the use of different immunohistochemistry (IHC) panels over time may lead to inconsistent immunohistochemistry markers for GBNEC diagnosis.

## 5 Conclusion

The presence of 30% GBNEC may not necessarily be the best tool for the differentiation of GBNEC and GBMiNEN. The presence of malignant tumor components may be more important in providing valuable prognostic information on survival for patients with mixed tumors. Patients with either pure or mixed GBNEC, displaying advanced tumor stages and lymphovascular invasion were at an independently increased risk of worse OS and RFS. Due to the small sample size of the study, the conclusions drawn above need to be validated by multicenter large-sample studies.

## Data availability statement

The raw data supporting the conclusions of this article will be made available by the authors, without undue reservation.

## Ethics statement

This study was conducted in accordance with the Declaration of Helsinki and in compliance with the study protocol and ethical guidelines for medical and health research involving human subjects. The study was approved by the institutional review committee of West China Hospital, Sichuan University (approval code 2021-445). A summary of the protocol of the study is available on the website of the hospital. The studies were conducted in accordance with the local legislation and institutional requirements. Written informed consent for participation was not required from the participants or the participants’ legal guardians/next of kin in accordance with the national legislation and institutional requirements.

## Author contributions

Y-FH: Writing – original draft. J-KW: Writing – original draft, Resources, Writing – review & editing. W-JM: Resources, Writing – review & editing. H-FG: Resources, Writing – review & editing. H-JH: Resources, Writing – review & editing. FL: Resources, Writing – review & editing. T-RL: Resources, Writing – review & editing. S-QY: Resources, Writing – review & editing. Y-SD: Resources, Writing – review & editing. R-QZ: Resources, Writing – review & editing. F-YL and Y-WJ: Writing – review & editing.
